# Supplementary figures and images for: MiRNA Differences Related to Treatment-Resistant Schizophrenia
Source: Int J Mol Sci. 2023 Jan 18;24(3):1891. doi: 10.3390/ijms24031891 (PMC9916039; doi:10.3390/ijms24031891)

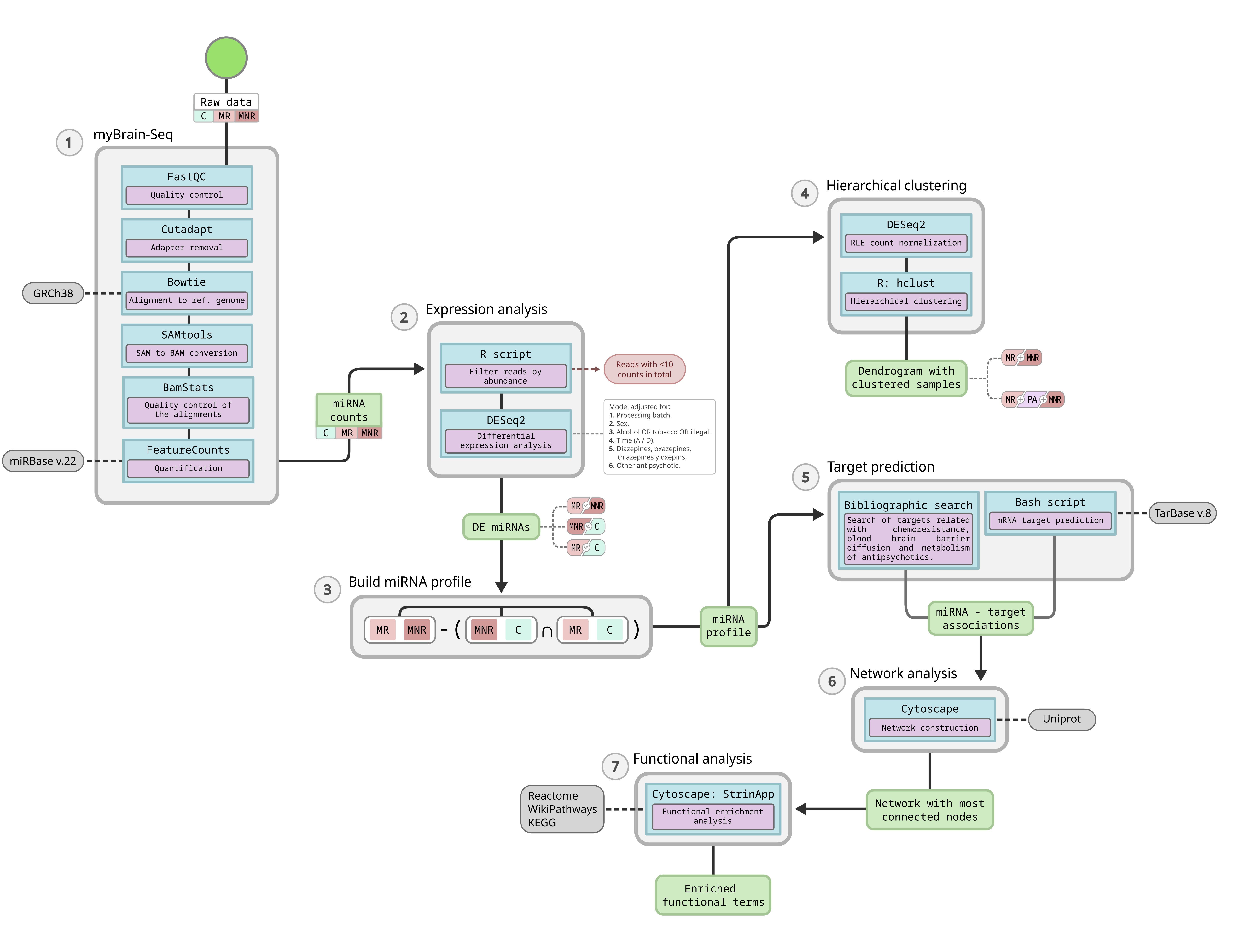

Supplement: Supplementary file 1 [file ijms-24-01891-s001.zip › supplementary Figure S1.png]
